# Editorial: Schwann cells: From their development to their clinical application

**DOI:** 10.3389/fncel.2022.1043419

**Published:** 2022-10-06

**Authors:** Kirsten Haastert-Talini

**Affiliations:** ^1^Institute of Neuroanatomy and Cell Biology, Hannover Medical School, Hanover, Germany; ^2^Center for Systems Neuroscience (ZSN), Hanover, Germany

**Keywords:** Schwann cells, functional recovery, primary cell culture, nerve injury, transplantation, spinal cord injury

The Research Topic entitled “*Schwann cells: From their development to their clinical application*” is focused on a better understanding of Schwann cell (SC) development, myelination, and their regeneration promoting repair phenotype. Gain of knowledge about diverse aspects of SC biology is of high interest, due to the peripheral glia cells key role for the orchestration of peripheral nerve maintenance and repair. Over the last decades research on SC development, myelination and their repair phenotype has revealed more insights in their functionality but therapy of severe peripheral nerve injuries is still far from being optimal. Especially in the clinical context, translational and direct knowledge about the functionality of SCs after transplantation into peripheral nerve or spinal cord injuries has not yet fully completed the picture of the therapeutic value of primary or stem cell derived human SCs. Furthermore, pharmaceutical strategies are still to be defined for increasing the therapeutic potential of human SC transplantation.

In total 42 authors from five different countries and three continents contributed to the current Research Topic with original research reports, different type of reviews, and one perspective. All the contributions are discussed in the following. They shed light on the recent challenges and developments from different viewpoints thereby adding to the aforementioned broad picture of SC biology and its use for purposes in regenerative medicine.

Sundaram et al. utilized embryonic dissociated dorsal root ganglia (DRG) cultures for the definition of the timeline of developmental SC stages as they transit from one to the next stage in this widely used co-cultures systems. They could demonstrate that SC precursor to immature SC transition occurs in mouse DRG/SC co-cultures and that with supplementation of ascorbic acid further differentiation from immature to mature SC stage can be induced. They confirmed the developmental processes by the analysis of the expression profiles of a number of relevant SC developmental genes as well as by myelination assays.

More original research reports included into the current Research Topic focus on the behavior of SCs after peripheral nerve injury. The article of Zhou et al. conducted *in vivo* and *in vitro* studies revealing a regeneration promoting function of the drug Epothilone (EpoB). Systemic administration of EpoB to rats for 7 days after sciatic nerve crush lesion accelerated the speed of axonal regeneration, remyelination and functional recovery *in vivo*. The authors further demonstrated that treatment with EpoB does not affect viability of neonatal rat SC cultures, with regard to cell cycle and apoptosis rate. Interestingly, the authors demonstrated that treatment EpoB significantly increased autophagy related markers as well as migration in neonatal rat SC cultures. The authors conclude that these events could be key for the EpoB effect they observed *in vivo*. Also the work published by Chen et al. was motivated by the link between successful peripheral nerve regeneration and SC behavior and signaling after injury. The authors utilized a mouse sciatic nerve transection model for exploring specifically the role of fibroblast growth factor 5 (FGF5) in this scenario. Molecular and histological expression analyses revealed an upregulation of FGF5 in the transected distal mouse sciatic nerve *in vivo*. Subsequent experiments using rat neonatal primary SC cultures demonstrated that FGF5 has a role as autocrine regulator of SC migration and adhesion *in vitro* and that this role is rendered *via* an upregulation of N-cadherin in SCs.

The mini review provided by Acheta et al. sets again in focus the role of SCs in the peripheral nerve regeneration process. More specifically, the authors reviewed the effect of therapeutic administration of low-intensity ultrasound (LIU) on repair SC behavior and expression profiles of pro-inflammatory cytokines or neurotrophic proteins in pre-clinical rodent studies. With their contribution to the current Research Topic, the authors light the need for comprehensive mechanistic studies on the effects of LIU for stimulating future clinical trials on it. The systematic review authored by Vallejo et al., in line with the request for comprehensive translational research, summarizes the results and conclusions from *in vivo* studies that were published from 2008 to 2022 and have used SC transplantation approaches for nerve gap repair. The article gives clear indication about the past, present and future of SC transplantation and delivers an encouraging outlook for new technologies to come using SC exosomes instead of complete cells in a future clinical setting. The perspective article provided by Errante et al., shares with us pre-clinical and clinical evidence from the authors' years-long work using SC transplantation within guidance conduits for nerve gap repair. Along with discussing techniques and results from pre-clinical work in rodents, the authors critically discuss valuable sources and methods for procuring sufficiently large autologous human SC populations for their use in suitable nerve guides.

The last, but not least, contribution included into this Research Topic, is the review written by Monje et al. who critically discuss current knowledge on human SC transplantation approaches for repairing the injured spinal cord. With this, it becomes even more evident how powerful the SC repair machinery could become, when optimally supported, even for nerve repair processes in the central nervous system. So there is another intriguing dimension in the regenerative medicine field that needs to be considered when thinking on the clinical application of human SCs. The authors clearly identify prospects and challenges for this specific kind of cell therapy by addressing safety issues and the question of alternative SC sources. By reading this article, the reader will, once again, be stimulated to define comprehensive future work for answering still outstanding questions and for developing techniques that can make a difference in the future.

Thank you to all authors for their valuable contributions!

## Author contributions

The author confirms being the sole contributor of this work and has approved it for publication.

## Conflict of interest

The author declares that the research was conducted in the absence of any commercial or financial relationships that could be construed as a potential conflict of interest.

## Publisher's note

All claims expressed in this article are solely those of the authors and do not necessarily represent those of their affiliated organizations, or those of the publisher, the editors and the reviewers. Any product that may be evaluated in this article, or claim that may be made by its manufacturer, is not guaranteed or endorsed by the publisher.

